# Evaluation of Surface Properties and Separation Performance of NF and RO Membranes for Phthalates Removal

**DOI:** 10.3390/membranes13040413

**Published:** 2023-04-06

**Authors:** En Qi Lim, Mei Qun Seah, Woei Jye Lau, Hasrinah Hasbullah, Pei Sean Goh, Ahmad Fauzi Ismail, Daryoush Emadzadeh

**Affiliations:** 1 Faculty of Chemical and Energy Engineering, Universiti Teknologi Malaysia, Skudai 81310, Johor, Malaysia; 2 Advanced Membrane Technology Research Centre (AMTEC), Universiti Teknologi Malaysia, Skudai 81310, Johor, Malaysia; 3 Department of Chemical and Biological Engineering, University of Ottawa, 161 Louis Pasteur, Ottawa, ON K1N 6N5, Canada

**Keywords:** phthalates, commercial membrane, nanofiltration, reverse osmosis, water

## Abstract

Many studies indicated that phthalates, a common plasticizer, lurk silently in water bodies and can potentially harm living organisms. Therefore, removing phthalates from water sources prior to consumption is crucial. This study aims to evaluate the performance of several commercial nanofiltrations (NF) (i.e., NF3 and Duracid) and reverse osmosis (RO) membranes (i.e., SW30XLE and BW30) in removing phthalates from simulated solutions and further correlate the intrinsic properties of membranes (e.g., surface chemistry, morphology, and hydrophilicity) with the phthalates removal. Two types of phthalates, i.e., dibutyl phthalate (DBP) and butyl benzyl phthalate (BBP), were used in this work, and the effects of pH (ranging from 3 to 10) on the membrane performance were studied. The experimental findings showed that the NF3 membrane could yield the best DBP (92.5–98.8%) and BBP rejection (88.7–91.7%) regardless of pH, and these excellent results are in good agreement with the surface properties of the membrane, i.e., low water contact angle (hydrophilicity) and appropriate pore size. Moreover, the NF3 membrane with a lower polyamide cross-linking degree also exhibited significantly higher water flux compared to the RO membranes. Further investigation indicated that the surface of the NF3 membrane was severely covered by foulants after 4-h filtration of DBP solution compared to the BBP solution. This could be attributed to the high concentration of DBP presented in the feed solution owing to its high-water solubility (13 ppm) compared to BBP (2.69 ppm). Further research is still needed to study the effect of other compounds (e.g., dissolved ions and organic/inorganic matters that might be present in water) on the performance of membranes in removing phthalates.

## 1. Introduction

Water pollution caused by plastic has become a serious concern with the development of the plastic industry. In 2018, 359 million tonnes of plastic were produced worldwide, and it is estimated that by 2025, a total of 250 million tonnes of plastics will be discharged [1]. Phthalates have been the well-known plasticizers in the industry since the 1930s due to their high compatibility with polyvinyl chloride (PVC). Apart from being plasticizers, they also act as additives in industrial products such as pesticides, paints, cosmetics, and electrical insulators. Researchers have categorized phthalates as a part of emerging pollutants (EPs) as they can persist for a long time in the environment, accumulate and spread from one species to another via the food chain [2,3].

Phthalates (a.k.a. phthalic acid) are the esters of 1,2-dibenzene dicarboxylic acid derived from a benzene ring and two ester groups [4]. They are transparent or slightly yellowish in appearance and odorless. These features make them difficult to be monitored in the environment, especially water bodies. In general, phthalates show good solubility in most organic solvents but are barely soluble in water. Moreover, they are less volatile and react with other chemical substances. Table 1 shows the physicochemical properties of the six common phthalates, which are di-2-ethylhexyl phthalate (DEHP), diisononyl phthalate (DINP), diisodecyl phthalate (DIDP), di-n-octyl phthalate (DnOP), dibutyl phthalate (DBP), and butyl benzyl phthalate (BBP). Compared to other phthalates, DBP and BBP have a much higher water solubility level and, thus, are selected in this work for investigation. As reported by the United States Environmental Protection Agency (USEPA), phthalates have been found in food and in humans [5]. Several studies reported the case of feminization in male animals and birth defects, especially amphibians, after being exposed to phthalates [6,7]. Additionally, phthalates are one of the endocrine disruptors which bring several negative long-term effects on human reproductive, neurological, and developmental systems [8].

Phthalates have undoubtedly brought a lot of convenience to society, but their harmful effects on humans and ecosystems cannot be ignored. A tidal wave of new research has documented their wide-ranging negative health impacts [16]. Emission of phthalates frequently happens as phthalates themselves do not bond covalently to the polymers, thus making them easily enter the environment via water, soil, and air. Phthalates could enter the environment in many ways, such as being lost in the form of solid waste or wastewater in the manufacturing process, diffusing into the atmosphere due to low volatility, and leaching from produced products [4]. Exposure to phthalates easily happens among the population of humans and animals via inhalation, ingestion, and absorption (skin contact).

To minimize exposure rates and meet stricter environmental regulations, several wastewater treatment technologies, including biodegradation, adsorption, and membrane technology, are being considered/used to remove phthalates [17]. Among these methods, membrane technology offers high efficiency for eliminating phthalates with less energy consumption, small space required, and a simple working principle [18]. It can also work together with other technologies, such as adsorption, to enhance its efficiency. There are four types of pressure-driven membranes which are microfiltration (MF, Pore size: 100 nm–2 μm), ultrafiltration (UF, Pore size: 2–100 nm), nanofiltration (NF, Pore size: 1–10 nm) and reverse osmosis (RO, Pore size: <1 nm) [19]. Each of them has different operating pressures, characteristics, and functions.

Currently, there is very little research on using pressure-driven membranes to remove phthalates. Based on the statistics obtained from Scopus, it is shown that there are only about 400 articles mentioning “membranes” and “phthalates” in the article texts over the past five years (years 2018–2022) (Search within Article title, Abstract, Keywords). Owing to the small molecular weight of phthalates (<450 g/mol), most of the researchers used NF or RO membranes to achieve the desired separation rate. In 2004, Bodzek et al. [20] investigated the performance of RO membrane (RO-DS3SE), NF membrane (NF-DS5DK), and UF membrane (UF-DSGM) in removing DEP, DBP, and DEHP from water at different operating pressures (20 bar for RO and NF membranes and 3 bar for UF membrane). Their findings indicated that the NF-DS5CK membrane showed the best phthalates removal performance, recording 99.9% retention for all three phthalates samples [20]. Even though the pore size of the UF membrane is larger than phthalates, this membrane could still record a relatively high removal rate against DEP and DEHP. The authors attributed the results to possible adsorption and/or partial membrane blocking [20].

On the other hand, Ozay et al. [21] conducted an experiment to investigate the efficiency of NF90 and NF90 + BW30 hybrid membrane processes in removing micropollutants and phthalates. The results showed that as high as 99.9% removal could be achieved by the hybrid membrane process (NF90 + BW30) in removing DEP and DEHP. The NF90 membrane, meanwhile, only achieved 97.7% and 98.9% for DEP and DEHP, respectively. Another study found that the NF90 membrane could achieve a higher phthalate (DMP, DEP, and DBP) rejection than NF270 membrane due to its smaller pore sizes [22]. The authors also reported that the fouled membranes tended to increase the phthalate removal efficiency because of the increase in sieving effect and surface hydrophobicity [22].

By taking into consideration the harmful effects of phthalates on humans and ecosystems, the main objective of this work is to evaluate the performance of several commercial membranes (NF and RO) in removing two types of phthalates with high water solubility levels under different testing conditions. All these commercial membranes are composed of PA selective layer supported by a microporous substrate. The cross-linking degree and pore size of the PA layer, however, varies depending on the synthesized conditions used in manufacturing each membrane. We will correlate the surface properties of membranes with the rejection rate of phthalates during the filtration process and investigate how the deposition of phthalates on the membrane surface affects the separation efficiency. Four commercially available membranes are selected (2 NF and 2 RO) for the removal of DBP and BBP at different pH, and the membrane surface characterization is carried out using a series of analytical instruments.

## 2. Materials and Methods

### 2.1. Materials

Four commercial thin film composite (TFC) membranes, including two RO (i.e., SW30XLE and BW30 from DuPont FilmTec^TM^, Wilmington, DE, USA) and two NF membranes (i.e., NF3 from RisingSun Membrane Technology (Beijing) Co., Ltd., China and Duracid from Suez (GE)) were used in this research study. Dibutyl phthalates (DBP) and benzyl butyl phthalates (BBP) with 99% and 98% purity, respectively, were purchased from Sigma-Aldrich, Saint Louis, MI, USA and were used to prepare stock solutions. Sodium sulfate (Na_2_SO_4_, Merck Sdn Bhd, Selangor, Malaysia) and sodium chloride (NaCl, Merck Sdn Bhd, Selangor, Malaysia) were used for the salt rejection test, while sulfuric acid (H_2_SO_4_, Merck Sdn Bhd, Selangor, Malaysia) and sodium hydroxide (NaOH, Merck Sdn Bhd, Selangor, Malaysia) were used for solution pH adjustment. All the solutions were prepared by using water (ASTM Type III) produced by a water purification system (Merck Millipore, Burlington, MA, USA).

### 2.2. Membrane Filtration Performance

**Figure 1:**
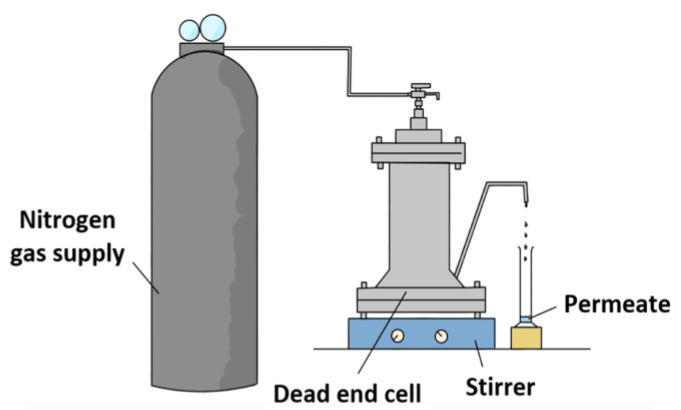
Schematic diagram of dead-end permeation cell setup for membrane evaluation.

Dead-end high-pressure filtration cell (HP4750, Sterlitech, Auburn, WA, USA) made of 316 stainless steel material, as illustrated in Figure 1, was used to carry out the experiments to determine the membrane filtration performances with respect to pure water permeability and salt/phthalates rejection. Prior to the filtration, the respective membrane was cut into circular coupon with an effective surface area of 14.60 cm^2^. The membrane coupons were then soaked in pure water and kept at 4 °C until testing. Each membrane was pre-compacted using nitrogen gas for 30 min at a pressure of 11 and 16 bar for NF and RO membranes, respectively, using pure water at a stirring speed of 300 rpm. This compaction is necessary in order to achieve steady-state flux. Then, the pressure was, respectively, reduced to 10 and 15 bar for NF and RO membranes. The measurement was started after 15-min operation at the specific pressure.

The water flux, J_W_ (L/m^2^·h), and pure water permeability, PWP (L/m^2^·h·bar) of each membrane can be evaluated using Equations (1) and (2), respectively.

(1) $$J_{W}=\frac{V}{A\times t}$$

(2) $$\mathrm{PWP}=\frac{J_{W}}{P}$$

where V is volume of permeate collected (L), A is membrane surface area (m^2^), t is taken to collect the permeate (h), and P is operating pressure (bar).

After the PWP test, salt rejection (R_S_) of each membrane was investigated by using either 1000-ppm Na_2_SO_4_ or 1000-ppm NaCl solution. Salt concentration in both feed and permeate was measured by using a benchtop conductivity meter (4520, Jenway, London, UK). Salt rejection, R_S_ (%), was calculated using Equation (3).

(3) $$R_{S}=\left|\frac{C_{\mathrm{initial}}-C_{\mathrm{final}}}{C_{\mathrm{initial}}}\right|\times 100$$

where $C_{\mathrm{initial}}$ and $C_{\mathrm{final}}$ are the concentration of salt in the feed and permeate, respectively.

The phthalates rejection test was studied using 10 ppm of DBP or 2 ppm of BBP as feed. 5 mL of permeate was collected for both tests, and the time was recorded. The rejection performance of each membrane was examined by using UV-vis spectrophotometer (DR5000, Hach Malaysia Sdn Bhd, Kuala Lumpur, Malaysia). The absorbance of both feed and permeate was measured by scanning them at the peak absorbance wavelength, i.e., 225 nm for DBP and 275 nm for BBP. The rejection of membrane against phthalate, R_P_ (%), can be determined using the following equation.

(4) $$R_{P}=\left|\frac{{\mathrm{abs}}_{\mathrm{initial}}-{\mathrm{abs}}_{\mathrm{final}}}{{\mathrm{abs}}_{\mathrm{initial}}}\right|\times 100$$

where ${\mathrm{abs}}_{\mathrm{initial}}$ and ${\mathrm{abs}}_{\mathrm{final}}$ are the absorbance of the feed and permeate, respectively.

Single 10 ppm of DBP solution and 2 ppm of BBP solutions were prepared by spiking 10 μL of DBP and 2 μL of BBP into 1000 mL of pure water, respectively. Amount of phthalates added is based on their solubility (DBP: ~13 mg/L and BBP: ~2.69 mg/L) in water. The stock solutions were then sonicated for 7 h in an ultrasonic bath (3800, Branson, Connecticut, USA). To further analyze the performance of the membranes in removing the phthalates, the effect of solution pH was also studied. The pH of the stock solutions (~pH 6) was adjusted to pH 10 and 3 using 1 M of NaOH solution or 0.1 M of H_2_SO_4_ solution, respectively. The pH value was measured using multiparameter-pH meter (PC2700, Eutech, Singapore). Lastly, the stability and durability of the membrane in removing the phthalates was conducted by running for a duration of 4 h. During the experiment, the flux and rejection were measured and recorded every 30 min.

An adsorption test was also performed to identify the ability of the NF3 membrane to adsorb the phthalates without applying a driving force. In this test, the membrane was placed within a dead-end filtration cell and subject to 2-h stirring at 500 rpm without having external driving force. The absorbance of the single DBP and BBP solutions in the cell before and after adsorption test was then measured to calculate the reduction of phthalates due to membrane adsorption.

### 2.3. Membrane Characterization

Attenuated total-reflectance Fourier transform infrared spectroscopy (ATIR-FTIR) (Nicolet iS10, Thermo Fisher Scientific, Waltham, MA, USA) was applied to identify the functional group of the membranes at wavenumber ranging 500–4000 cm^−1^. An average of 32 scans was performed to yield the spectrum of each membrane. Surface roughness of the dried membranes was determined using atomic force microscopy (AFM) (NX10, Park Systems, Suwon, Republic of Korea). The hydrophilicity of membranes was evaluated via the sessile drop method using contact angle goniometer (OCA15Pro, Data Physics, San Jose, CA, USA). At least 10 measurements were performed on each membrane sample in order to yield the average value. A water droplet (pure water) of 0.5 μL was placed on the dried membrane surface for each measurement. To visualize the change of membrane surface before and after the filtration process, scanning electron microscopy (SEM) (S-3400N, Hitachi, Tokyo, Japan) was used. Prior to SEM analysis, the samples were sputter-coated with a thin layer of gold so better images could be produced.

## 3. Results and Discussion

### 3.1. Surface Chemistry and Surface Morphology of Membranes

**Figure 2:**
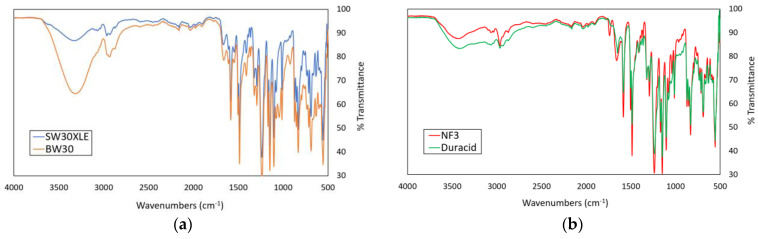
The ATR-FTIR spectra of different membranes, (**a**) SW30XLE and BW30 (RO type) and (**b**) NF3 and Duracid (NF type).

Figure 2a,b shows the FTIR spectra of the commercial RO and NF membranes used in this work, respectively. The results indicated that the commercial TFC RO and NF membranes used were composed of a polyamide (PA) layer due to the presence of characteristics peaks of the PA layer, including C-N stretching (1583 and 1584 cm^−1^) and amide I and C=O stretching (1647, 1659, 1660, and 1662 cm^−1^). Comparing between two RO membranes, it was found that the BW30 membrane exhibited higher intensity than the SW30XLE membrane for the broad peak that appeared in the region of 3000–3500 cm^−1,^ and this was mainly due to its lower PA crosslinking degree, which produced higher amount of –OH groups [23]. For the NF membranes, it was found that both NF3 and Duracid membranes displayed very similar peaks, except an additional peak at 1737 cm^−1^ was detected in the NF3 membrane. The additional peak could be due to the presence of carbonyl groups (C=O) resulting from the use of different additives during PA layer synthesis. It must be pointed out that the exact synthesis conditions are remained largely unknown to the public mainly due to the trade secret in manufacturing the membrane products.

**Figure 3:**
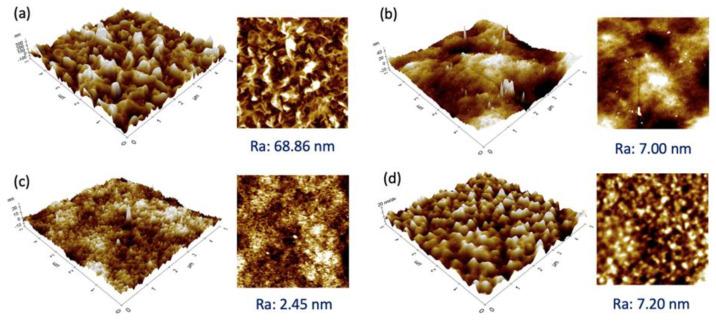
3D (left) and 2D (right) AFM images of RO and NF membranes together with R_a_ value, (**a**) SW30XLE, (**b**) BW30, (**c**) NF3, and (**d**) Duracid.

Figure 3 presents the 3D and 2D images of the selective layer of the membranes studied. Generally, all the membranes exhibited a relatively smooth surface with a R_a_ value < 10 nm, except for the SW30XLE membrane with a significantly rougher surface (R_a_: 68.86 nm). Our findings agreed with the work of Ebrahim et al. [24], in which the researchers also reported a very similar roughness value of the SW30XLE membrane, i.e., 78 nm. From the figure, the SW30XLE and Duracid membranes display typical nodular morphologies with obvious hills and valleys. However, the Duracid membrane has a significantly lower R_a_ value than the SW30XLE membrane. The roughness value of the BW30 membrane was similar to the Duracid membrane, even though their surface morphology was completely different. Among the membranes studied, the NF3 membrane displayed the lowest surface roughness, i.e., 2.45 nm. It must be noted that surface roughness will not only affect the membrane water flux but also govern the solutes deposition/adsorption on the membrane surface. The rougher surface was reported to have a higher fouling tendency as it provides larger contact areas for solutes to deposit [25,26].

### 3.2. Pure Water Permeability, Contact Angle, and Salt Rejection of Membranes

**Figure 4:**
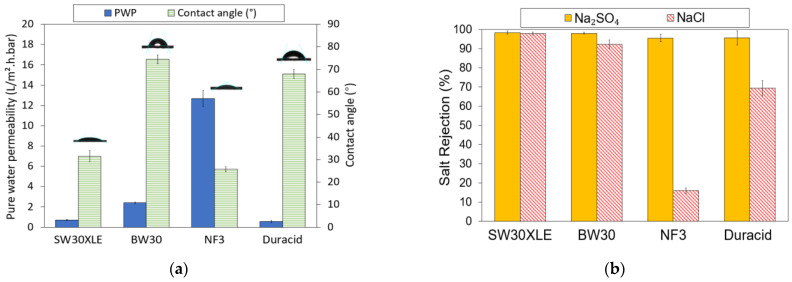
Properties of RO and NF membrane, (**a**) Pure water permeability (PWP) and water contact angle and (**b**) Monovalent and divalent salt rejection.

The PWP and water contact angle of four different types of commercial TFC membranes are presented in Figure 4a. As can be seen, the water contact angle on the membrane is in the order of BW30 (74.46°) > Duracid (67.97°) > SW30XLE (31.50°) > NF3 (25.72°). The water contact angle is correlated with the hydrophilicity of the TFC membrane, where the smaller the contact angle, the higher the surface hydrophilicity. A membrane with greater surface hydrophilicity will allow more water to pass through its matrix and thus results in higher water flux. However, the pore size of the membrane will also play a role in determining the membrane water flux in addition to surface hydrophilicity. For instance, the water contact angle of SW30XLE is the second lowest among the membrane studies, but such good hydrophilicity does not really contribute to high water permeance as its dense structure is the dominant factor governing water transport. Our findings indicated that all the TFC membranes exhibit hydrophilic characteristics as their water contact angles are <80°.

With respect to PWP, it is obvious that the NF3 membrane recorded the highest value (12.69 L/m^2^.h.bar). Its promising water permeance could be attributed to its lowest water contact angle (compared to other membranes) and larger surface pore size than the RO-type membranes [27]. Due to its high PWP, the NF3 membrane requires less operational energy and system footprint to achieve the same water productivity as other membranes. Additionally, its greater surface hydrophilicity could play a role in minimizing the foulants deposition on its surface [28,29]. The lowest PWP of the Duracid membrane could be attributed to its denser selective layer due to the addition of sulfonyl chloride (–SO_2_Cl) on the PIP-TMC-based membrane [29,30]. Furthermore, the support layer of the Duracid membrane was reported to be significantly thicker compared to other NF membranes (DK, DL, and NF270) tested in the work of Gao et al. [29]. Its support layer is around two times thicker than the support of typical NF membranes. This thick structure tends to create greater resistance for water to transport and thus leads to lower water flux.

With respect to the salt rejection, as shown in Figure 4b, it can be observed that all the TFC membranes exhibited promising Na_2_SO_4_ rejection (95.5–98.3%). In comparison, both RO membranes (SW30XLE and BW30) performed better than the NF membranes (NF3 and Duracid) in removing Na_2_SO_4_, recording at least 97.9% rejection. Moreover, the SW30XLE and BW30 membranes also achieved significantly higher NaCl rejection of 97.9% and 92.3%, respectively. This is because the RO membranes are designed for the purposes of brackish/seawater desalination process and aim to purify the water by rejecting the passage of monovalent salt. Compared to the BW30 membrane, the SW30XLE membrane demonstrated better performance in removing dissolved ions owing to its higher degree of PA cross-linking, which formed a denser skin layer. On the other hand, the NF membranes showed lower salt rejection rates compared to the RO membrane. This was mainly due to the larger surface pore size, which was not effective in retaining a small hydrated radius of Na and Cl ions [31].

### 3.3. Membrane Performance for Phthalates Removal

#### 3.3.1. Membrane Rejection against DBP and BBP

**Figure 5:**
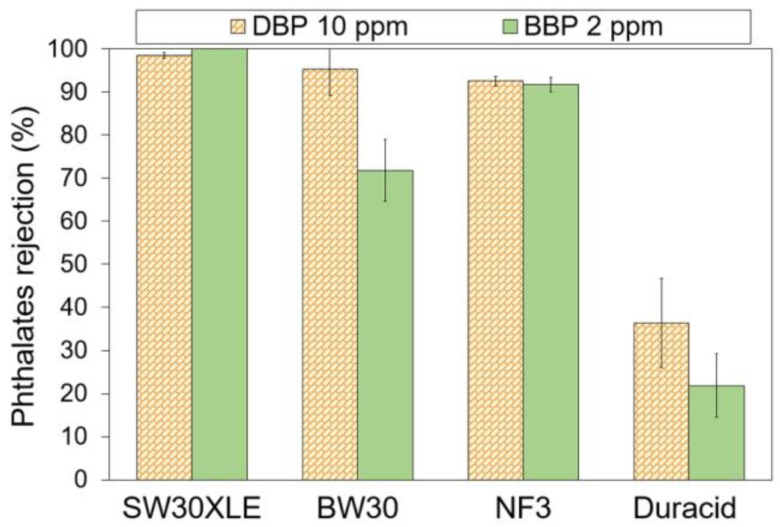
Phthalates rejection by different RO and NF membranes at ~pH 6.30.

Figure 5 shows the performance of commercial TFC membranes for the removal of two different types of phthalates. The experiment was separately conducted using DBP and BBP solution with pH at ~6. Experimental findings showed that both SW30XLE and NF3 membranes were able to achieve a relatively constant rejection against both DBP and BBP. However, upon comparing to the NF3 membrane, the SW30XLE membrane demonstrated a significantly higher rejection against DBP (98.5%) and BBP (100%). The excellent performance could be attributed to the high cross-linking degree of the polyamide layer.

The SW30XLE membrane Is a typical m-phenylene diamine–trimesoylchloride (MPD-TMC) membrane with a full aromatic polyamide structure due to the absence of a dominant peak at 1730 cm^−1^ [32]. This selective layer offers promising rejection against small solutes, including monovalent ions, while allowing water to flow through [33]. Higher PA crosslinking reduces the gap space between polymer chain segments so that the segment vibration is limited, thereby improving selectivity [34]. Additionally, a highly cross-linked membrane tends to have a reduced swelling effect under neutral conditions due to high mechanical strength [34]. Hence, its structure, especially the pore size, will not be easily altered.

On the other hand, the NF3 membrane is a piperazine–trimesoylchloride (PIP-TMC) based membrane with relatively good surface hydrophilicity. Its water contact angle is the lowest one compared to the rest of the membranes tested in this work. Since the log K_ow_ of both phthalates are between 4.50 and 4.73, these chemicals are typically considered semi-hydrophobic. The hydrophilic (membrane)-hydrophobic (phthalates) interaction, in addition to the small pore size of the membrane, could create repulsion, which is beneficial to reject phthalates. Nevertheless, the rejection recorded by the NF3 membrane is lower than the SW30XLE membrane, and this is mainly due to its larger surface pore size, which is mainly used to separate divalent salt instead of monovalent salt [35]. With respect to membrane surface charge, this factor might not play a significant role in influencing phthalate removal as both DBP and BBP do not really carry charge at neutral conditions.

Compared to the SW30XLE and NF3 membranes, the performance of the BW30 membrane in rejecting phthalates is inconsistent as it records a high DBP rejection (95.3%) but a low BBP rejection (71.8%). The reduced separation efficiency of the BW30 membrane compared to the SW30XLE membrane is due to its looser polyamide layer, as the membrane is designed to desalinate brackish water instead of seawater. Furthermore, our findings showed that the Duracid was not suitable for treating phthalates-contaminated solutions owing to its extremely low phthalates separation rate, recording 36.4% and 21.9% for DBP and BBP, respectively. Based on the membrane specifications from the manufacturer [36], the Duracid membrane is suitable to be applied to purify acid-containing wastewater and concentrate metals at low pH streams instead of removing organic pollutants in a neutral environment.

#### 3.3.2. Effect of pH on DBP and BBP Removal

**Figure 6:**
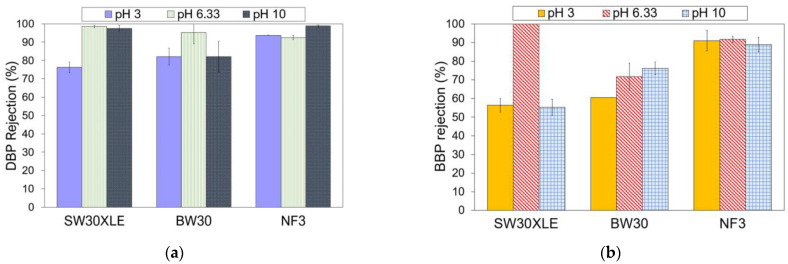
Effect of pH on the membrane performance for the removal of (**a**) DBP and (**b**) BBP.

Industrial wastewater typically has varying pH depending on the types of contaminants, chemicals, and microorganisms present in the solution. Therefore, it is also important to study to what extent the solution pH could affect the membrane performance in removing phthalates. Figure 6 presents the membrane performance in rejecting DBP and BBP at different pHs. In this section, only three membranes (i.e., SW30XLE, BW30, and NF3) were studied. We excluded the Duracid membrane from the investigation because of its extremely low phthalates rejection, as reported in the previous section.

As shown in Figure 6, the phthalates rejection performance of the NF3 membrane was relatively higher than the SW30XLE and BW30 membrane at pH 3 and 10. Additionally, it was observed that the NF3 membrane also exhibited more consistent phthalates removal at a wide-range pH condition than other membranes. A study revealed that the NF3 membrane (made of PIP-TMC) exhibited high chemical stability at pH between 2 and 11 [37]. Our experimental findings indicated that the NF3 membrane was able to remove >90% of DBP and 88% of BBP at a pH ranging from 3 to 10. Conversely, the performances of both SW30XLE and BW30 membranes were negatively affected when they were used in acid (pH 3) and alkali (pH 10) conditions. It is believed that the NF3 membrane, which is made of PIP-TMC, could be more suitable than the MPD-TMC-based RO membranes for phthalates removal owing to the better hydrophilic (membrane)-hydrophobic (phthalates) interaction resulted from its lowest water contact angle.

In general, the DBP rejection was adversely affected when the feed solution was acidic (pH 3). A low pH condition could hydrolyze DBP into protonated phthalic acid, which increases the number of proton charges in DBP [38]. Because of this, the rejection of the membrane against DBP tends to decrease owing to the increase in attractive force between the positively charged DBP and the negatively charged membranes, increasing the passage of DBP through the membrane.

#### 3.3.3. Quality of Treated Water

**Figure 7:**
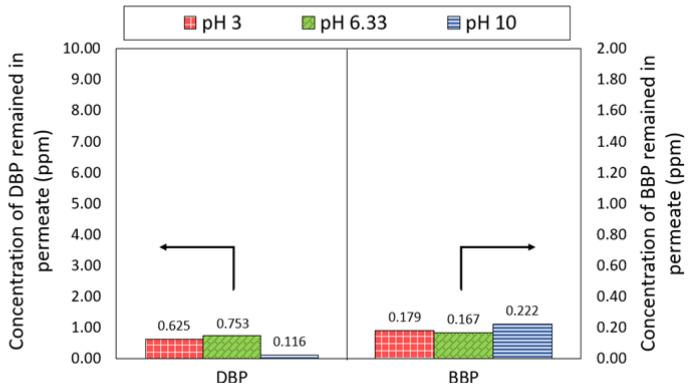
Concentration of DBP and BBP in the permeate of NF3 membrane.

Figure 7 compares the concentration of DBP and BBP in the permeate produced by the NF3 membrane at different pH. A significant decrease in the DBP concentration in the feed solution was observed, where the concentration was reduced from 10 ppm to 0.753 ppm at ~pH 6.33. While under extreme acid and alkali conditions, the NF3 membrane was also able to achieve DBP concentrations of 0.625 ppm and 0.116 ppm, respectively. With respect to the BBP, the NF3 membrane could attain a very low concentration of the phthalate, recording at 0.179–0.222 ppm. The detection of lower BBP concentration compared to the DBP concentration was mainly due to the low feed concentration of BBP used as a result of its low water solubility. It was found that the NF3 membrane was capable of producing a permeate with DBP and BBP concentrations lower than the threshold limit value (5 ppm) set by the United States Environmental Protection Agency (USEPA) for phthalates [39].

### 3.4. Short-term Performance Stability of NF3 Membrane

**Figure 8:**
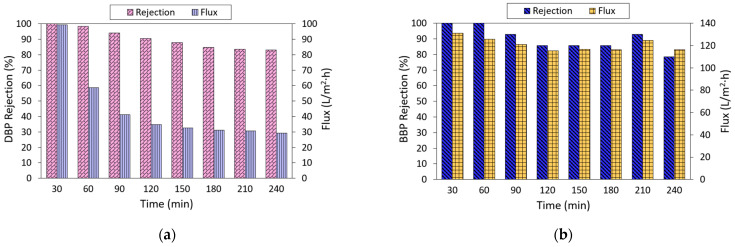
Performance stability of NF3 membrane as a function of time in filtrating (**a**) DBP (10 ppm) and (**b**) BBP (2 ppm) solution at pH ~6.3.

The performance of the NF3 membrane against DBP and BBP removal was further evaluated as a function of filtration time, and the results are presented in Figure 8. As shown, the rejection of the NF3 membrane tended to decrease as a function of time. Its DBP rejection decreased from almost 100% at the early of the filtration process to 83.1% at the end of the experiment. In comparison, the rejection of membrane against BBP also decreased, but its performance was approximately stable after a 120-min operation. With respect to water flux, the membrane displayed decreasing flux in filtering both DBP and BBP. However, the membrane suffered from more severe flux deterioration in filtering DBP compared to the filtration of BBP. The decrease in membrane water flux could be due to surface fouling, while the decrease in phthalates could be partly due to the adsorption behavior of the membrane. It is believed that besides the sieving and charge effects, the adsorption of phthalates onto the membrane itself was also contributing to the separation. Once the membrane’s adsorption against phthalates is near saturation, more phthalates are likely to permeate through the membrane, which leads to lower rejection. Based on the findings obtained from 2-h adsorption, it was found that the NF3 membrane could reduce the phthalate concentration in water by 17.74% and 47.3% for DBP and BBP, respectively.

**Figure 9:**
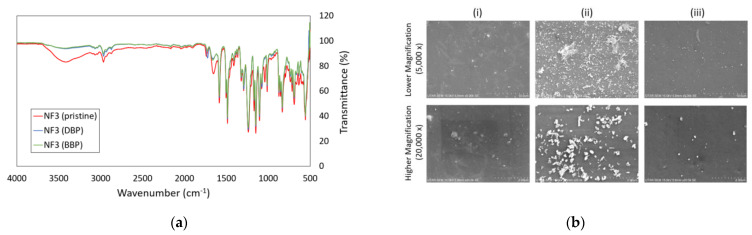
(**a**) FTIR spectrum of pristine NF3 membrane before and after DBP and BBP filtration tests and (**b**) SEM surface images of (i) Pristine NF3 membrane, (ii) NF3 membrane used for DBP filtration and (iii) NF3 membrane used for BBP filtration at two magnifications (5000× and 20,000×).

Based on the FTIR spectra shown in Figure 9a, it was obvious that several peaks of the pristine NF3 membrane were altered after it was used for DBP and BBP filtration. The intensities of peaks at 3000–3500 cm^−1^ region and ~1737 cm^−1^ were reduced, and this was mainly due to the membrane surface fouling caused by the deposition/adsorption of phthalates. The SEM images in Figure 9b indicated that the surface of the used membranes (for DBP and BBP removal) was deposited with particles, and significantly large particles were detected on the surface of the membrane used for DBP removal. The findings were in good agreement with the significant flux decline in the membrane used for DBP removal compared to the one used for BBP removal. Even though the feed concentration of phthalates was low for the experiment, the low solubility of DBP and BBP in water could easily cause them to form particles, especially on the membrane surface. This can be explained by the fact that when the membrane rejects phthalates during the filtration process, the accumulation of phthalates on the membrane surface could lead to a significant increase in phthalates concentration, exceeding its solubility. Because of this reason, phthalates can form particles on the membrane surface and causes fouling.

Comparing the SEM surface image of the membrane used to treat DBP-containing water, the membrane used for BBP filtration did not have much fouling on its surface. Less fouling caused by BBP could be due to its lower water solubility (2.69 ppm) than that of DBP (13 ppm). In this study, DBP and BBP solutions were prepared by spiking 10 µL of pure DBP and 2 µL of pure BBP chemicals into 1-L RO water, respectively. Obviously, the amount of BBP particles that are left in the filtration cell would certainly be fewer than DBP particles during the membrane filtration process.

## 4. Conclusions

In this work, we evaluated the performance of four commercial TFC membranes in removing two selected phthalates from an aqueous solution and correlated the surface properties of the membrane with removal efficiency. Although all the membranes were made of a polyamide layer supported by a substrate, the membrane surface properties varied depending on the synthesis conditions of each manufacturer. Among the four membranes tested, our results showed that the NF3 membrane was the most promising membrane by taking into account its high DBP (92.5–98.8%) and BBP rejection (88.7–91.7%) regardless of solution pH. This membrane could also purify phthalates-contaminated water with a wide pH range (3–10) to a safe level as stipulated in the USEPA regulations (<5 ppm). Furthermore, the NF3 membrane also exhibited the highest water permeance compared to the rest of the membranes. The excellent filtration characteristics of the NF3 membrane could be attributed to its good surface hydrophilicity coupled with a looser skin layer and its negative charge feature. However, further investigation indicated that the surface of the NF3 membrane was severely covered by foulants after 4-h filtration of DBP solution compared to the BBP solution. This could be attributed to the high concentration of DBP presented in the feed solution owing to its high-water solubility (13 ppm) compared to BBP (2.69 ppm). It must also be pointed out that further research is still needed to study the effect of other compounds (e.g., dissolved ions, organic/inorganic matters, etc., that might be present in water) on the performance of membranes in removing phthalates.

## Figures and Tables

**Table 1:** Physicochemical properties of the six common phthalates.

Phthalates	Chemical Formula	Molar Mass (g/mol)	Melting Point (°C)	Boiling Point (°C)	Relative Density (Water = 1.0) (g/cm^3^)	Solubility in Water (mg/L)	Ref.
Di-2-ethylhexyl phthalate	C_24_H_38_O_4_	390.6	−46	384	0.986	0.270	[9,10]
Diisononyl phthalate	C_26_H_42_O_4_	418.6	−48	252 (at 5 mm Hg)	0.980	0.200	[9,11]
Diisodecyl phthalate	C_28_H_46_O_4_	446.7	−46	253 (at 4 mm Hg)	0.960	0.280	[9,12]
Di-n-octyl phthalate	C_24_H_38_O_4_	390.6	−25	220 (at 2 mm Hg)	0.978	0.022	[9,13]
Dibutyl phthalate (DBP)	C_16_H_22_O_4_	278.4	−35	340	1.050	11.200	[9,14]
Butyl benzyl phthalate (BBP)	C_19_H_20_O_4_	312.4	−35	370	1.100	2.690	[9,15]

## Data Availability

Data sharing is not applicable.
